# Aqueous Humor Cytokine Profile in Primary Congenital Glaucoma

**DOI:** 10.3390/jcm12093142

**Published:** 2023-04-26

**Authors:** Carlos Oribio-Quinto, Barbara Burgos-Blasco, Pilar Pérez-García, Laura Espino-Paisán, Beatriz Sarriá, José Ignacio Fernández-Vigo, Julian García-Feijóo

**Affiliations:** 1 Ophthalmology Department, Hospital Clínico San Carlos, Institute of Health Research (IdISSC), 28040 Madrid, Spain; bburgos171@hotmail.com (B.B.-B.); perezgarciapilar.ppg@gmail.com (P.P.-G.);; 2 Laboratorio de Investigación en Genética de Enfermedades Complejas, Instituto de Investigación Sanitaria del Hospital Clínico San Carlos (IdISSC), 28040 Madrid, Spain; laurep80@gmail.com; 3 Department of Metabolism and Nutrition, Institute of Food Science, Technology and Nutrition (ICTAN), Spanish National Research Council (CSIC), 28040 Madrid, Spain; 4 Departamento de Inmunología, Oftalmología y ORL, Facultad de Medicina, Universidad Complutense de Madrid, 28232 Madrid, Spain

**Keywords:** glaucoma, primary congenital glaucoma, cytokines, aqueous humor

## Abstract

Background: Cytokine profile in patients with primary open-angle glaucoma (POAG) differs from that in healthy controls. Due to the different pathophysiological mechanisms involved in the genesis of primary congenital glaucoma (PCG) and POAG, it is possible that the cytokine profile could also differ. The main objective of this study was to compare the concentrations of cytokines in the aqueous humor of patients with PCG with those of POAG patients and a control group. Methods: A cross-sectional study was conducted. Aqueous humor samples were taken from PCG and POAG patients eligible for glaucoma or cataract surgery and from patients undergoing cataract surgery. Twenty-seven cytokines were analyzed using the Human Cytokine 27-Plex Immunoassay Kit (Bio-Rad Laboratories, Hercules, CA, USA). Results: A total of 107 subjects were included: patients with PCG (*n* = 19), patients with POAG (*n* = 54), and a control group (CG) of patients undergoing cataract surgery (*n* = 34). Most cytokines measured in aqueous humor in PCG presented decreased values compared with POAG and controls. A statistically significant difference was observed in IL-1ra, IL-2, IL-5, IL-7, IL-8, IL-10, IL-12, IL-15, IL-17A, Eotaxin, FGF basic, G-CSF, GM-CSF, IFN-γ, MIP-1α, PDGF-bb, MIP-1β, RANTES, TNF-α, and VEGF. Conclusion: PCG patients have a cytokine profile in aqueous humor different from POAG patients and patients without glaucoma, characterized by lower concentrations of multiple cytokines.

## 1. Introduction

Glaucoma is a heterogeneous group of diseases that commonly involve the loss of retinal ganglion cells and their axons, which results in a characteristic alteration of the optic nerve and a corresponding pattern of vision loss [1]. Childhood glaucoma can be classified into primary and secondary glaucoma, which include primary congenital glaucoma (PCG) and juvenile open-angle glaucoma. The latter includes glaucoma following cataract surgery, glaucoma associated with non-acquired systemic diseases or syndromes, glaucoma associated with non-acquired ocular anomalies, and glaucoma associated with acquired conditions [2,3].

PCG is the most common form of pediatric glaucoma, accounting for approximately 18% of childhood blindness [2,4]; its incidence varies geographically [5,6,7] and affects more than 300,000 children globally [8]. Although most PCG cases are sporadic, up to 40% follow an autosomal recessive inheritance pattern with variable penetrance [2,7]. Four loci (GLC3A on 2p21, GLC3B on 1p36, GLC3C, and GLC3D) have been identified as associated with PCG, and PCG-causing mutations have been identified in genes within two of the four loci [2]. Cytochrome P450 1B1 (CYP1B1) mutations were discovered within the GLC3A locus and are the most commonly known cause of PCG [9].

Altered cytokine expression in the aqueous humor has been described in numerous ophthalmological conditions, including age-related macular degeneration, diabetic retinopathy, and uveitis. These cytokines may play a role in both the pathogenesis and progression of these conditions [10].

In glaucoma, models of retinal ganglion cell death have shown that inflammatory responses linked to ischemia and hypoxia and characterized by the upregulation of cytokine concentration can directly link intraocular pressure (IOP) with diminishing numbers of these cells [11]. Moreover, as apoptotic reactions play a key role in glaucomatous neurodegeneration, the role of certain cytokines linked to oligodendrocyte death and subsequent retinal ganglion cell apoptosis highlights the importance of cytokine expression and regulation in the pathogenesis and progression of glaucoma [12,13].

It has been shown that the cytokine profile in the aqueous humor of patients with primary-different types of glaucoma, including primary open-angle glaucoma (POAG) and pseudoexfoliative glaucoma, differs from that in healthy controls. These studies have found a distinct profile characterized by an increased expression of proinflammatory and immunoregulatory cytokines, including tumor necrosis factor (TNF)-a and vascular endothelial growth factor (VEGF), interleukin (IL)-6, and IL-8 [10] and have suggested that they could be used as biomarkers [14,15,16]. Additionally, a correlation has been described between the levels of proinflammatory cytokines and the severity of glaucomatous neuropathy found in POAG patients [17].

Due to the different pathophysiological mechanisms involved in the genesis of PCG and POAG, it is possible that cytokine expression in the aqueous humor could also differ. However, to this date, there is no data regarding the profile of cytokines present in aqueous humor patients with PCG.

Therefore, the main objective of this study was to compare the concentrations of different cytokines in the aqueous humor of patients with PCG with those of POAG patients and a cohort of cataract patients with no other ophthalmological diseases.

## 2. Materials and Methods

This cross-sectional study was conducted at the Clínico San Carlos Hospital in Madrid. Patients diagnosed with PCG who required surgery were recruited for the study. Patients diagnosed with POAG and candidates for surgery (cataract or glaucoma) were also recruited.

Data from 29 cataract patients and 27 patients with POAG from a previous study performed at the same center that used the same methodology were added to our sample. Patients who underwent cataract surgery with no other ocular diseases were included in the control group (CG).

The inclusion criteria for the PCG group were a diagnosis of PCG, being a candidate for glaucoma surgery, and no prior ocular surgeries or ophthalmologic diseases. The diagnosis of PCG was made following the Childhood Glaucoma Research Network Classification and was defined as pediatric patients with elevated IOP levels, defined as an IOP of 21 mmHg or more. Morphological alterations such as increased optic nerve cupping, focal notching, or cup-to-disc asymmetry of 0.2 or more between both eyes, the presence of corneal changes such as increased corneal diameter or Haab striae, or visual field defects consistent with optic nerve glaucomatous damage [8,18].

The inclusion criteria of the POAG group were age > 40 years, a patient candidate for cataract or glaucoma surgery, and diagnosis of POAG. The diagnosis of POAG was made according to the definition of the European Glaucoma Society and required the presence of a progressive optic neuropathy with characteristic morphological changes at the optic nerve head, retinal nerve fiber layer, and visual field alterations in the absence of other ocular disease or congenital anomalies.

The inclusion criteria of the CG were age > 40 years, no previous ocular surgeries, and absence of concomitant ophthalmological pathology (other than cataract). All patients in the CG had to be candidates for cataract surgery at the moment of inclusion.

The exclusion criteria for all groups were the history of ocular surgery other than cataract surgery, cataract surgery or an ocular laser procedure in the six months prior to inclusion in the study, another type of glaucoma other than chronic PCG or POAG, concomitant ophthalmological pathology, and concomitant topical treatment other than anti-glaucomatous eye drops were excluded.

At the time of inclusion, the following variables were noted for each patient: demographic data, previous and current medical treatments, and past medical history. On the day of the surgery, an aqueous humor sample of approximately 40–50 μL was collected using a 30- G anterior chamber cannula attached to a 1-mL syringe under aseptic conditions as the first step of the surgery. This was done previous to the introduction of any substance in the anterior chamber and without contacting the iris or any intraocular structure. The samples were stored at −80 °C until analysis.

Cytokine concentrations in aqueous humor samples were determined by the Bio-Plex Pro Human Cytokine 27-Plex Immunoassay kit (Bio-Rad Laboratories, Hercules, CA, USA). This technique is performed on a platform composed of fluorescent magnetic surfaces that allows the simultaneous detection of up to 96 samples. With this technology, it is possible to quantify up to 27 cytokines in a single analysis. The cytokines quantified in this study were: interleukin (IL)-1b, IL-1ra, IL- 2, IL-4, IL-5, IL-6, IL-7, IL-8, IL9, IL-10, IL-12, IL-13, IL-15, IL-17, eotaxin, basic fibroblast growth factor (FGF-b), granulocyte colony-stimulating factor (G-CSF), granulocyte–macrophage colony-stimulating factor (GM-CSF), interferon–gamma (IFN-γ), interferon–gamma-induced protein (IP-10), monocytic chemotactic protein (MCP-1), macrophage inflammatory protein (MIP)-1a, MIP-1β, platelet-derived growth factor (PDGF)-bb, regulated on activation, normal T cell expressed and secreted (RANTES), tumor necrosis factor (TNF)-a, and vascular endothelial growth factor (VEGF). The limit of detection of this kit that refers to the concentration of analyte on the standard curve for which signal value is above the background control value plus 2-fold of its standard deviation varies for each individual cytokine and has a range of 0.6–6.4 pg/mL; the upper limit of quantification has a range of 836–95,484 pg/mL and varies depending on the cytokine analyzed.

For the sample analysis, 50 μL of each were dispensed into the microwells of each platform and read with a Luminex MAGPIX reader (Luminex Corporation, Austin, TX, USA).

The fluorescence intensity of each sample was compared against their standard curve, and the cytokine concentration was interpolated. The Bio-Plex ManagerTM software (Bio-Rad Laboratories, Hercules, CA, USA) was used to calculate the concentrations of the 27 cytokines.

The data was processed and analyzed statistically using IBM SPSS Statistics for Mac, version 25.0 (IBM Corp., Armonk, NY, USA). For categorical variables, frequency distributions were calculated and compared using Fisher’s test. Quantitative variables are presented as means and standard deviations. To analyze the differences in the concentrations of each cytokine between the different subgroups, a Mann–Whitney U test was performed, and the Bonferroni correction for multiple comparisons was applied. The correlation between the two parameters was determined by Spearman correlation. A *p*-value < 0.05 was considered to be statistically significant.

## 3. Results

A total of 107 subjects were included in this study, which was distributed into the following groups: patients diagnosed with PCG (PCG, *n* = 19), patients diagnosed with POAG (POAG, *n* = 54), and CG (CG, *n* = 34).

Demographic and clinical data for each group are summarized in Table 1.

All patients in the control group underwent cataract surgery. In the POAG group, 27 patients had a trabeculectomy, 14 had cataract surgery, and 13 received a Preserflo Microshunt implant. In the PCG group, eight patients had a goniotomy performed, 10 had a trabeculectomy, and one had an Ahmed valve. Three patients in the PCG group tested positive for CYP1B1 gene mutations.

Most of the cytokines measured in aqueous humor patients with PCG were lower than those in patients with POAG and CG. When comparing the three groups, a statistically significant difference was observed in IL-1ra, IL-2, IL-5, IL-7, IL-8, IL-10, IL-12, IL-15, IL-17A, Eotaxin, FGF basic, G-CSF, GM-CSF, IFN-g, MIP-1α, PDGF-bb, MIP-1β, RANTES, TNF-α, and VEGF (all, *p* < 0.05). The concentrations and significant differences of these cytokines are detailed in Table 2.

The degree of correlation and statistical significance of each analyzed cytokine are summarized in Table 3.

The concentration of IL-6 was positively correlated with age (r = 0.477; *p* = 0.039), whereas VEGF was negatively correlated with age (r = −0.469; *p* = 0.043). As for IOP, IL-6 and IL-13 showed a positive correlation (r = 0.518; *p* = 0.028 and r = 0.604; *p* = 0.008); while IL-5, IL-12, and PDGF-bb were negatively correlated (r = −0.529; *p* = 0.024, r = −0.493; *p* = 0.037 and r = −0.523; *p* = 0.026). Only IFN-γ showed a significant correlation with the number of topical medications (r= −0.482; *p* = 0.036).

## 4. Discussion

The main objective of our study was to obtain a profile of cytokines present in the aqueous humor of PCG patients and to compare it to that of POAG patients and a control group of patients undergoing cataract surgery. Our results reveal a decreased concentration in all of the 27 cytokines analyzed when compared to POAG and CG.

The cytokine profile in the aqueous humor of patients with POAG has been analyzed by several research groups [10,16,17,19]. These studies have found augmented levels of several cytokines, including IL-8, which is involved in neutrophil chemotaxis, and TNF-α, which has been postulated to be involved in the apoptosis of retinal ganglion cells [16,20,21]. The cytokine levels in patients with POAG reported by other authors are in agreement with those found in our study.

On the other hand, there is no data on the cytokine environment present in the aqueous humor of PCG patients, although it has been studied in other ocular conditions. Lai et al. studied the cytokine profile in the aqueous humor of 13 children with congenital cataracts (mean age 3.89 ± 2.34 years) compared with senile cataract patients; of the 22 cytokines analyzed, 11 had lower concentrations in the congenital cataract group, these included GM-CSF, IL-10, IL-2, IL-3, IL-4, IL-5, IL-6, IL-8, and TNF-α. Furthermore, a significant correlation between several of these cytokines and age was observed; specifically, MCP-1 and IL-8 were negatively correlated with age in children with congenital cataracts [22]. In our study, the concentrations of GM-CSF, IL-10, IL-2, IL-5, IL-6, IL-8, and TNF-α were also significantly diminished in the PCG group. The concentration of IL-6 was positively correlated with age, whereas VEGF was negatively correlated with age. It is possible that age is an important factor in cytokine levels; however, it is difficult to prove this hypothesis in the present study.

In a cohort of children with uveitis, Sijssens et al. investigated the aqueous humor cytokine profile and found higher concentrations of IL-1, IL-2, IL-4, IL-6, IL-8, IL-10, IL-12 p-70, IL-13, IL-18, IFN-γ, TNF-α, soluble intercellular adhesion molecule 1 (sICAM-1), soluble vascular cell adhesion molecule 1 (sVCAM-1), RANTES, Eotaxin (CCL11), and interferon-inducible 10-kDa protein (IP-10; CXCL10) in children compared with adolescents and adults [23]. The results of Sijssens et al. contrast our findings. This could be due to uveitis being a more inflammatory disease, as well as differences in pathogenic mechanisms. Nevertheless, they reveal a distinct age-related disease profile. Our results showed that IL-6 and VEGF levels significantly correlated with age (Table 3). Although it is possible that age plays a role in the distinct profile found in our study, it is difficult to confirm this using our current data.

The type of glaucoma and its distinct pathogenesis could play a role in the profile of cytokines found in aqueous humor. Previous studies have found elevated levels of IL-9, IL-12, IFN-α, IFN-γ, monokine induced by interferon–gamma (MIG or CXCL9), and IL-10 in POAG patients; these cytokines play a role in Th1-mediated proinflammatory response and leukocyte activation [10]. The concentrations of IL-6, IL-8, VEGF, and TNF-α have been found to be higher in uveitic glaucoma patients than in POAG patients, and these were correlated with the inflammatory response observed in slit-lamp examination [24], and the profile found in POAG is different of that found in primary open-angle glaucoma, with the latter having increased levels of IL-8 and CXCL9 [10]. Therefore, in addition to age-driven differences in concentrations, the lower concentration of cytokines found by our study in PCG patients could be explained by the pathogenic mechanisms involved in this subtype of glaucoma, in which the developmental dysgenesis, mainly the presence of angle alterations that result in thickened trabecular beams and compression that produce aqueous humor outflow obstruction, plays a more significant role than inflammation [25].

Additionally, our findings could also be explained by a mechanism of cytokine depletion, which has been described by Sahay et al. in the later stages of pseudoexfoliative glaucoma, in which patients have a diminished cytokine concentration probably due to exhaustion of the inflammatory response [26]. However, this explanation seems unlikely as the patients included in our PCG group did not show advanced disease at the time of recruitment.

Although cytokine levels in aqueous humor of healthy children are unknown, blood levels may provide insights into age-related trends. Decker et al. investigated cytokine blood levels in healthy children under 12 years. In unstimulated samples, a decrease in cytokine concentration with age was observed for IL-1ra, IP-10, and TNF-α. However, in stimulated samples, an increase in IL-4, IFN-γ, and TNF-α levels with age was found for all stimulatory conditions [27]. Intracellular detection of cytokines in children’s blood showed a significant positive correlation between age and intracellular cytokine levels of IFN-γ, IL-2, IL-4, and TNF-α in CD4+ cells, as well as for IFN- γ and TNF- α in CD8+ cells. However, this trend was not observed in adults [28]. A thorough analysis of cytokine concentrations in the blood of 72 patients was performed by Kleiner et al. [29]. The production of some molecules (IL-1ra, IL-2, IL-7, IL-8, IL-9, IL-10, IL-12, IL-16, basic FGF, G-CSF, M-CSF, GM-CSF, IP-10, MCP-1, MIP-1*α*, RANTES, and VEGF) was constant throughout development, whereas IL-17 and eotaxin increased with age. In contrast, MIP-1*β*, IL-18, GRO-*α*, MIF, SCF, SCGF-*β,* IL-2R*α*, SDF-1*α*, and TRAIL were reduced in adult samples. Immune maturation has been postulated as a possible explanation for the age-related increase in cytokine secretion in children. Care should be taken when interpreting these findings, given that they show cytokine expression in healthy subjects. Nevertheless, although these studies cannot be used to explain the behavior of cytokine production in glaucoma patients, they further support the hypothesis of an age-related trend that can modify cytokine expression and profile regardless of disease and could help understand the variations observed in our results.

Several limitations in the current study should be acknowledged, including the relatively low number of samples in the PCG group. Larger samples are difficult to obtain, given that it is a relatively rare disease that not many centers treat; thus, even with the low number of samples, this study provides useful insight into the cytokine profile found in these patients.

Another limitation is the lack of an age-matched control group, which is difficult to obtain for ethical reasons. A solution to this limitation could be to obtain a control group of patients with congenital cataracts that could provide an age-matched cohort; although these patients cannot be considered healthy subjects, this could provide further information relative to the profile observed in this study.

Nevertheless, this is the first study to investigate the cytokine profile in aqueous humor of PCG patients and compare it with POAG and a control group of cataract patients. Previous studies have revealed a link between different cytokines and clinical data, such as intraocular pressure levels [30], and a correlation between higher concentrations of several cytokines and disease progression [31]. Hence, it would be interesting for future studies to investigate the correlation between cytokine levels found in PCG patients and variables such as disease severity, genetic profile, and progression, or success rates of different surgeries.

## 5. Conclusions

In conclusion, this study evaluated the cytokine profile found in the aqueous humor of PCG patients and compared it to that of POAG and cataract patients without other ophthalmological diseases. Our results show that PCG patients have a distinct cytokine profile in the aqueous humor, which is characterized by lower concentrations of multiple cytokines. Although the explanation for this difference is unclear, this lower concentration could be influenced by differences inherent to the age group of the participants or by the distinct pathogenic mechanisms that result in the development of PCG that differ from the ones involved in POAG.

## Figures and Tables

**Table 1:** Patients’ demographic and clinical data.

	Control Group			POAG			PCG		
	Mean	SD	Range	Mean	SD	Range	Mean	SD	Range
Age (years)	77.63	9.31	58–93	74.00	8.88	49–92	3.72	4.68	1–11
IOP (mmHg)	15.14	2.22	10–18	20,2	5.18	15–35	32.25	12.45	18–45
Topical treatment (n)	0.00	0.00	0.00	2.18	0.84	1–3	1.21	0.79	1–2

POAG: primary open-angle glaucoma; PCG: primary congenital glaucoma; IOP: intraocular pressure.

**Table 2:** Concentrations and statistical comparison of cytokines in aqueous humor of PCG, POAG, and control groups.

Cytokines	LOD (pg/mL)	Control Group			POAG Group			PCG Group			Comparison (CG/POAG/PCG)	Post Hoc	Post Hoc
											Kruskal–Wallis	PCG/CG	PCG/POAG
		Mean (pg/mL)	SD	Range (pg/mL)	Mean (pg/mL)	SD	Range (pg/mL)	Mean (pg/mL)	SD	Range (pg/mL)			
IL-1b	0.6	0.70	1.83	0–8.86	0.68	1.31	0–5.18	0.07	0.15	0–0.65	0.087		
IL-1ra	5.5	1326.11	2824.95	0–13,564.38	6914.38	29,275.3	0–207,035.2	276.79	512.76	0–2127.88	0.030 *	0.143	0.025 *
IL-2	1.6	1.16	3.57	0–20.4	2.67	6.15	0–28.34	0.18	0.43	0–1.44	0.002 *	0.028 *	0.001 *
IL-4	0.7	0.16	0.35	0–2.01	0.37	0.85	0–4.28	0.11	0.16	0–0.54	0.337		
IL-5	0.6	13.61	23.39	0–86.64	34.24	46.65	0–196.8	19.62	53.07	0–230.57	0.023 *	0.282	0.019 *
IL-6	2.6	16.15	71.57	0–419.82	6.60	9.05	0–57.41	17.06	49.48	0–218.75	0.165		
IL-7	1.1	5.21	10.65	0–54.08	8.16	20.90	0–135.98	0.78	1.10	0–4.05	0.023 *	0.049 *	0.026 *
IL-8	1.0	53.66	131.60	0–509.08	110.60	396.40	0–2360.95	4.02	3.47	0–13.96	0.012 *	0.204	0.010 *
IL-9	2.5	17.20	50.90	0–248.96	19.79	70.31	0–412.07	3.45	7.66	0–32.84	0.253		
IL-10	0.3	8.31	20.72	0–96.21	15.35	27.95	0–119.37	0.88	1.57	0–7.06	0.005 *	0.162	0.004 *
IL-12	3.5	6.40	16.66	0–62	13.96	21.36	0–69.81	2.80	7.62	0–32.84	0.002 *	1.000	0.010 *
IL-13	0.7	0.88	3.08	0–18.1	1.19	2.48	0–11.43	0.28	0.34	0–1.17	0.294		
IL-15	2.4	272.96	684.56	0–2569.43	427.23	839.15	0–3406.22	3.42	10.42	0–38.26	0.021 *	0.030 *	0.034 *
IL-17A	3.3	12.63	30.50	0–139.87	13.19	27.24	0–169.62	0.81	1.99	0–8.72	0.001 *	0.004 *	0.002 *
Eotaxin	2.5	6.85	5.39	0–19.09	7.49	5.81	0–26	2.83	2.26	0–8.61	0.001 *	0.005 *	<0.001 *
FGF basic	1.9	10.31	18.36	0–67.4	15.97	54.79	0–385.02	1.13	3.39	0–11.1	0.009 *	0.185	0.007 *
G-CSF	1.7	116.68	260.36	0–1019.52	232.09	356.24	0–1482.36	6.56	15.05	0–64.61	<0.001 *	<0.001 *	<0.001 *
GM-CSF	2.2	3.69	13.73	0–73.26	2.23	8.17	0–53.34	0.15	0.45	0–1.73	0.050 *	0.967	0.063
IFN-g	6.4	25.40	49.53	0–230.41	33.84	58.10	0–266.33	1.33	2.18	0–10	<0.001 *	<0.001 *	<0.001 *
IP-10	6.1	10,133.6	27,679.3	0–112,266	16,082.3	54,665.8	0–359,972.8	154.98	136.20	0–518.3	0.089		
MCP-1 (MCAF)	1.1	207.26	122.23	0–429.27	195.99	155.70	0–501.85	150.04	80.15	0–290.1	0.289		
MIP-1a	1.6	1.19	1.97	0–10.62	3.36	10.66	0–77.43	0.16	0.31	0–1.34	<0.001 *	<0.001 *	<0.001 *
PDGF-bb	2.4	42.52	124.65	0–665.52	57.83	108.25	0–441.59	0.88	2.09	0–5.58	0.003 *	0.012 *	0.003 *
MIP-1b	2.9	8.16	8.75	0–31.06	12.32	19.95	0–108.92	2.65	3.39	0–13.27	0.006 *	0.023 *	0.006 *
RANTES	1.8	63.05	182.86	0–1007.48	43.05	62.53	0–237.97	7.82	14.24	0–61.7	0.025 *	0.100	0.022 *
TNF-a	6.0	14.09	26.21	0–105.89	25.76	53.90	0–214.78	0.84	1.23	0–3.85	0.001 *	0.001 *	0.001 *
VEGF	3.1	826.60	2252.27	0–9216.14	993.76	2505.71	0–12,789.26	49.42	177.87	0–776.13	0.002 *	0.049 *	0.001 *

POAG: Primary open-angle glaucoma; CG: control group; PCG: primary congenital glaucoma; PIL: interleukin; FGF-b: basic fibroblast growth factor; G-CSF: granulocyte colony-stimulating factor; GM-CSF: granulocyte–macrophage colony-stimulating factor; IFN-γ: interferon–gamma; IP-10: interferon–gamma-induced protein; MCP-1: monocytic chemotactic protein; MIP: macrophage inflammatory protein; PDGF: platelet-derived growth factor; RANTES: regulated on activation, normal T cell expressed and secreted; TNF: tumor necrosis factor; VEGF: vascular endothelial growth factor; LOD: Lower limit of detection. Significance values have been adjusted using Bonferroni correction for multiple comparisons. * Statistically significant differences.

**Table 3:** Cytokine level correlations with age, IOP, and number of topical medications in the PCG group.

Cytokines	Age		IOP		Number of Topical Medications	
	r	*p*	r	*p*	r	*p*
IL-1b	−0.37	0.119	−0.316	0.202	−0.281	0.243
IL-1ra	−0.162	0.508	−0.309	0.213	−0.365	0.124
IL-2	−0.125	0.609	−0.193	0.443	−0.229	0.346
IL-4	−0.103	0.675	−0.138	0.585	−0.151	0.536
IL-5	−0.335	0.161	−0.529	0.024 *	−0.254	0.293
IL-6	0.477	0.039 *	0.518	0.028 *	−0.226	0.353
IL-7	0.271	0.262	0.359	0.144	0.159	0.515
IL-8	0.035	0.888	−0.065	0.797	−0.319	0.183
IL-9	−0.188	0.44	−0.114	0.653	−0.079	0.748
IL-10	−0.208	0.393	−0.413	0.089	−0.148	0.547
IL-12	−0.336	0.159	−0.493	0.037 *	−0.126	0.609
IL-13	0.261	0.28	0.604	0.008 *	0.365	0.124
IL-15	−0.37	0.119	−0.468	0.05	−0.09	0.714
IL-17A	−0.328	0.17	−0.37	0.13	−0.319	0.184
Eotaxin	−0.101	0.68	0.165	0.513	−0.178	0.466
FGF basic	−0.096	0.697	−0.261	0.295	−0.309	0.198
G-CSF	0.697	0.528	−0.023	0.929	0.09	0.09
GM-CSF	−0.172	0.481	−0.286	0.25	−0.083	0.735
IFN-γ	−0.189	0.439	0.25	0.396	−0.482	0.036 *
IP-10	0.24	0.323	0.368	0.133	0.026	0.915
MCP-1(MCAF)	−0.026	0.917	0.107	0.673	−0.21	0.388
MIP-1𝛼	0.062	0.801	0.269	0.28	−0.169	0.489
PDGF-bb	−0.432	0.065	−0.523	0.026 *	−0.208	0.394
MIP-1*β*	−0.045	0.855	0.161	0.522	−0.2	0.412
RANTES	−0.154	0.528	−0.028	0.913	0.009	0.972
TNF-𝛼	−0.364	0.125	−0.336	0.173	−0.328	0.17
VEGF	−0.469	0.043 *	−0.452	0.060	−0.306	0.203

IOP: intraocular pressure; PIL: interleukin; FGF-b: basic fibroblast growth factor; G-CSF: granulocyte colony-stimulating factor; GM-CSF: granulocyte–macrophage colony-stimulating factor; IFN-γ: interferon–gamma; IP-10: interferon–gamma-induced protein; MCP-1: monocytic chemotactic protein; MIP: macrophage inflammatory protein; PDGF: platelet-derived growth factor; RANTES: regulated on activation, normal T cell expressed and secreted; TNF: tumor necrosis factor ; VEGF: vascular endothelial growth factor; * Statistically significant differences.

## Data Availability

The data presented in this study are available on request from the corresponding author. The data are not publicly available due to privacy reasons.
